# Outcomes Definitions and Statistical Tests in Oncology Studies: A Systematic Review of the Reporting Consistency

**DOI:** 10.1371/journal.pone.0164275

**Published:** 2016-10-07

**Authors:** Romain Rivoirard, Vianney Duplay, Mathieu Oriol, Fabien Tinquaut, Franck Chauvin, Nicolas Magne, Aurelie Bourmaud

**Affiliations:** 1 Department of Medical Oncology Lucien Neuwirth Cancer Institute, Saint Priest en Jarez, France; 2 Department of Public Health, Hygée Center, Saint Priest en Jarez, France; 3 Inserm, Clinical Investigation Center 1408, 42055 Saint-Etienne, France; 4 Department of Radiation Therapy Lucien Neuwirth Cancer Institute, Saint Priest en Jarez, France; University of North Carolina at Chapel Hill School of Medicine, UNITED STATES

## Abstract

**Background:**

Quality of reporting for Randomized Clinical Trials (RCTs) in oncology was analyzed in several systematic reviews, but, in this setting, there is paucity of data for the outcomes definitions and consistency of reporting for statistical tests in RCTs and Observational Studies (OBS). The objective of this review was to describe those two reporting aspects, for OBS and RCTs in oncology.

**Methods:**

From a list of 19 medical journals, three were retained for analysis, after a random selection: British Medical Journal (BMJ), Annals of Oncology (AoO) and British Journal of Cancer (BJC). All original articles published between March 2009 and March 2014 were screened. Only studies whose main outcome was accompanied by a corresponding statistical test were included in the analysis. Studies based on censored data were excluded. Primary outcome was to assess quality of reporting for description of primary outcome measure in RCTs and of variables of interest in OBS. A logistic regression was performed to identify covariates of studies potentially associated with concordance of tests between Methods and Results parts.

**Results:**

826 studies were included in the review, and 698 were OBS. Variables were described in Methods section for all OBS studies and primary endpoint was clearly detailed in Methods section for 109 RCTs (85.2%). 295 OBS (42.2%) and 43 RCTs (33.6%) had perfect agreement for reported statistical test between Methods and Results parts. In multivariable analysis, variable "number of included patients in study" was associated with test consistency: aOR (adjusted Odds Ratio) for third group compared to first group was equal to: aOR Grp3 = 0.52 [0.31–0.89] (P value = 0.009).

**Conclusion:**

Variables in OBS and primary endpoint in RCTs are reported and described with a high frequency. However, statistical tests consistency between methods and Results sections of OBS is not always noted. Therefore, we encourage authors and peer reviewers to verify consistency of statistical tests in oncology studies.

## Introduction

In oncology, quality and methodology of published clinical studies are essential to support an informed decision making [1]. Since 1996 and publication of the Consolidated Standards of Reporting of Trials (CONSORT) statement [2], many productive efforts have been made to improve the quality of reporting for randomised controlled trials [3]. Currently, it exists many other reporting guidelines for enhancing the quality of a variety of study types: For example, The Strengthening the Reporting of Observational Studies in Epidemiology (STROBE) statement for observational studies [4], Preferred Reporting Items for systematic reviews and meta-Analyses (PRISMA) for systematic reviews and meta-analyses [5] and reporting recommendations for tumor mARKer prognostic studies (REMARK) for tumor marker studies [6]. The peer review system [7] is mandatory in almost all journals and improves the overall quality of reporting for publications [8].

Despite an increasing rate of publications analyzing the quality of reporting of studies in oncology [9], few systematic reviews have specifically analyzed the reporting of the outcomes definition. For interventional studies,, the item relating to the description of pre-specified primary and secondary judgment criteria measures is an essential methodological point in the CONSORT checklist [10]. For observational studies, the item concerning the definition of variables (outcomes, exposures, predictors, potential confounders and potential effect modifiers) is a critical methodological criteria in the STROBE checklist [4],: clear descriptions, and steps taken to adhere to them are particularly important for any disease condition which are of primary interest in the study. Moreover, an element of reporting evaluation has not been studied in previous reviews: the item entitled "Statistical methods". This item enables to assess if articles provide a clear and exhaustive description of statistical methods, including statistical tests. But no recommendation exist, either in the CONSORT or in the STROBE, that would advise to check if statistical tests used in Results section are consistent with those described in Methods. Discrepancies between the statistical tests described in methods and tests really performed in results can lead to bias and, so, interfere with interpretation of the results.

Therefore, the main objective of the present systematic review was to describe quality of reporting for primary outcome measure in oncology clinical trials and for variables of interest in oncology observational studies. For the second objective, we investigated characteristics of manuscripts associated with a perfect consistency for statistical tests.

## Methods

### Search strategy

A list of medical journals has first been developed, selected on the following criteria: Non-organ specific journals, which publish articles dealing with cancer. This list was divided into 3 groups: A first group of generalist journals with Impact Factor (IF) above 6, a second group of journals specialized in oncology with high IF (above 6 IF) and a third group of oncology journals with middle IF (above 4 IF) [11]. Selection of these journals was decided in a multidisciplinary way. The list of the 19 Journals was as follow: the first group made of Generalist journals: New England Journal of Medicine, Lancet, Journal of the American Medical Association, Annasl of Internal Medicine, Plos Med, British Medical Journal, JAMA internal Medicine; the second group made of oncology journals with an Impact Factor above 6: Cancer Research, Journal of Clinical Oncology, Lancet Oncology, Journal of National Cancer Institut, CA: A Cancer Journal for Clinicians, Annals of Oncology; the third group made of oncology journals with an Impact factor above 4: British Journal of Cancer, European Journal of Cancer, The Oncologist, Cancer, International Journal of Cancer, Journal of the National Comprehensive Cancer Network. Among the 19 journals the statistician (F. Tinquaut) conducted a random selection: For each group a number between 1 and the number of Journals contained in each group was assigned to each journal. A random number was then drawn by group (following a discrete uniform distribution between 1 and the maximal number per group). The numbered corresponding Journal was then selected. Archives of the three included journals (British Medical Journal (BMJ), Annals of Oncology (AoO) and British Journal of Cancer (BjC)) were searched so that to identify all original articles, published between March 2009 and March 2014. The last search was performed in February 2015 [12].

### Study selection

Observational studies or interventional studies in English-language, focusing on a particular subject related to oncology and published between March 2009 and March 2014 in BMJ, AoO or BjC journals were eligible for inclusion. The main outcome of the study was to be accompanied by a corresponding statistical test.

Exclusion criteria were: Phase I, II, or IV trials; descriptive studies, meta-analyses, systematic reviews, prediction model building studies, test validation studies, recommendations, letter to the editor, medico-economic studies and fundamental research studies. Studies based on censored data and / or having the survival as primary endpoints were excluded from the analysis for two reasons: first tests used (log-rank test, cox proportional hazards survival analysis) are very specific ones. Second, those studies are generated in a purpose of drug development and authorization: in this case, data produced are most carefully verified by safety administrations. Those controls are far beyond those performed in a simple publication process. So included them would bias the results. This subject should be reported on its own, elsewhere.

From identified articles, the screening consisted to realize a first articles selection, on title and on abstract. Then, the potentially eligible articles were selected on full text. Two independent readers (RR and VD) made separately a selection with those criteria. Then the two remaining selections were compared and articles with a conflicting selection results were reviewed by a third reader, a senior methodologist (AB).

### Data collection

One author (RR) extracted the following data from included studies and a second author (MO) checked the extracted data: Name of publishing journal, year of publication, number of patients included, cancer studied, study type, description of outcome measures in Methods (interventional studies), definition of variables in Methods (observational studies), description of statistical test related to main endpoint/variables in Methods, type and name of statistical test, same name of statistical test used for main endpoint/variables assessment in Results, as compared to Methods, name of this test in Results, significant results for main endpoint, concordant conclusion on the main endpoint result.

### Data measurement

Two variables were constructed to describe the quality of outcomes reporting: the first one is the “variables description” for the observational studies, the second one “primary outcome description” for interventional studies. For observational studies, the authors used STROBE recommendations [4] to carry out the scoring of the variable. For criterion "definition of variables in Methods", the authors noted the answer "yes" only if there was a descriptive and exhaustive definition of all the variables considered and included in the analysis, such as outcomes, exposures, predictors, potential confounders and potential effect modifiers. A descriptive and exhaustive definition of diagnostic criteria was also needed for obtaining a "yes" answer. In all other cases, the authors noted the "no" answer for this criterion. In Results section, authors recorded if a statistically significant association between disease and the exposures was reported.

Concerning interventional studies, authors relied on the CONSORT recommendations [10] to score the quality of reporting for primary endpoint. If all pre-specified outcome measures, were identified and exhaustively defined, the authors noted the answer "yes" to criterion "description of outcome measures in Methods". In all other situations, this criterion has been side "no" by the authors. It was also studied whether results for primary end point have met statistical significance.

Concerning consistency for statistical tests between Methods and Results parts in included studies, only one test per article was considered: it was the statistical test performed to assess the main endpoint of each study. First, all included studies were categorized into 3 groups in order to get a categorical variable: One group with perfect agreement for statistical test that authors considered as the most suitable methodology. A second group in which name of test was described in Methods section but not reminded in Results section, that was considered as an acceptable methodology. The third group included all articles with obvious discrepancy between the two sections, and was consequently considered as manuscripts with major methodological problem.

Secondly, a qualitative description of the causes of the discrepancies observed for the articles selected in the third group was made, in order to explore it.

### Statistical methods

For the first two objectives, variables collected and constructed were described with %, Frequency and number of missing data. For the third part of the analysis, in order to identify studies’ and Journals’ factors associated with a complete statistical test consistency, tests have been performed on a recombined categorical variable: All studies with a perfect consistency for statistical test were classified in a "1"group and all other studies were classified in a "0" group. Variables tested in the univariable analysis were: Name of the Journal, Year of publication, Sample Size, tumor Site, Type of Study, Concordant Conclusion on the Main EndPoint, Type of Test, Main EndPoint described in methods. Odds Ratios (OR), their 95% confidence intervals and two-sided P-values (Likelihood Ratio-test: LR-test) were estimated by conditional logistic regression. A multivariable model was planned to be realized with every available covariates, since there was only 8 of them, against 826 articles to be analyzed. Analyses were carried out with the R software, version 3.1.2 (http://www.R-project.org). α-risk was set at 5%. Significance level was set for p-values ≤ 0.05.

## Results

### Literature search

**Fig 1:**
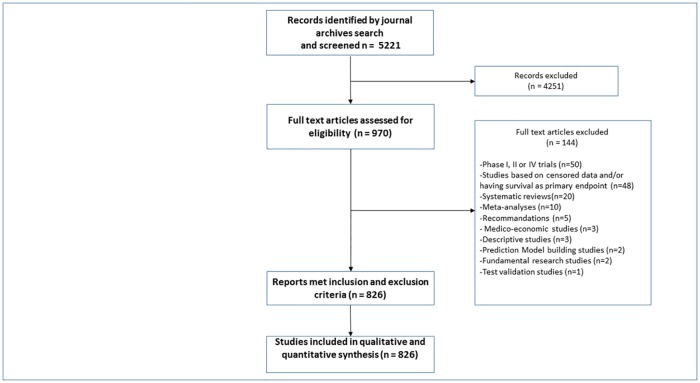
Flow chart: selection of studies process.

The search in BMJ, AoO, and BjC archives resulted in 5221 identified records. After title and abstract screening, 970 full text articles were assessed for eligibility. Finally, 826 studies met inclusion and exclusion criteria and were included in analysis (Fig 1).

### Characteristics of selected studies

Among the 826 studies included in systematic review (S1 file), 698 (84.5%) corresponded to observational studies and 128 (15.5%) were interventional studies, comprising 29 (3.5%) phase III randomized controlled trials. 401 (48.5%) of studies were published in *Annals of Oncology*, 378 (45.8%) in *British Journal of Cancer* and 47 (5.7%) in *British Medical Journal*. When only one tumor site was studied, main reported localizations were: Breast cancer (21.8% of studies), colon and/or rectum cancer (11.3% of studies), gynecological cancer (7% of studies, Table 1).

**Table 1:** Characteristics of Studies Included in Analysis (N = 826).

Characteristics	AoO (N = 401)	BjC (N = 378)	BMJ (N = 47)	Total, N (%)
Type of study				
Observational	334	326	38	698 (84.5)
Interventional	67	52	9	128 (15.5)
**Sample size, n° of patients**				
Median	331	960	4439	608
Interquartile range	119–1136	254–4569	1157–34352	160–2574
**Year of publication**				
2009	52	62	9	123 (14.9)
2010	62	61	15	138 (16.7)
2011	82	73	7	162 (19.6)
2012	112	72	7	191 (23.1)
2013	72	91	5	168 (20.3)
2014	21	19	4	44 (5.3)
**Tumor Site**				
**One site studied**	**302**	**308**	**35**	**645 (78.1)**
Breast	104	69	7	180 (21.8)
Colon/Rectum	33	54	6	93 (11.3)
Gynecological*	18	29	11	58 (7)
Leukemia/Lymphoma	35	17	0	52 (6.3)
Prostate	11	23	6	40 (4.8)
Lung	25	14	0	39 (4.7)
Stomach/Oesophagus	9	18	2	29 (3.5)
Renal	10	12	0	22 (2.7)
Pancreas	10	9	0	19 (2.3)
Head and Neck	9	9	0	18 (2.2)
Other	38	54	3	95 (11.5)
**Two or more sites studied**	**100**	**69**	**12**	**181 (21.9)**

AoO: Annals of Oncology; BjC: British Journal of Cancer; BMJ: British Medical Journal.

* Corresponds to the following sites: Cervix, Ovarian, Endometrium, Vagina, vulva.

### Reporting of variables and outcomes definitions

Variables were described in Methods section for all studies. A significant association between exposure and disease was observed for 618 studies (88.6%) in observational studies. Primary endpoint was clearly detailed in Methods section for 109 interventional studies (85.2% of cases). Result for the primary endpoint was statistically significant for 87 studies (68% of cases). A concordant conclusion on the main result was observed in 97.7% of cases.

### Reporting of statistical test

In observational studies, for the main analysis, test was parametric in 88.8% of cases (620 studies). Among these tests, logistic regression was the most used (416 studies, 59.6% of cases), followed by the chi-square test (70 studies, 10% of cases). Consistency of reporting for statistical test between methods and results section was evaluated as perfect for 295 studies (42.2% of cases). Name of test was not reminded in Results section for 385 studies (55.2% of cases) and an obvious discrepancy for test reporting was observed in 18 studies (2.6% of cases, Table 2).

**Table 2:** Reporting of variables and statistical test in observational studies (N = 698).

Reporting Item	AoO	BjC	BMJ	Total, N (%)
**Variables described in Methods**				
Yes	334	326	38	698 (100%)
No	0	0	0	0 (0%)
**Significant association disease/exposures**				
Yes	300	286	32	618 (88.6%)
No	34	39	6	79 (11.3%)
NA^1^	0	1	0	1 (0.1%)
**Concordant conclusion on the main result**				
Yes	329	320	37	686 (98.3%)
No	5	6	1	12 (1.7%)
**Test described in Methods**				
Yes	324	320	38	682 (97.7%)
No	10	6	0	16 (2.3%)
**Type of test**				
Parametric	284	298	38	620 (88.8%)
Nonparametric	40	22	0	62 (8.9%)
ND ^1^	10	6	0	16 (2.3%)
**Name of test**				
Logistic regression	173	217	26	416 (59.6%)
Chi-square	44	23	3	70 (10%)
Poisson regression	11	13	6	30 (4.3%)
Wilcoxon / Kruskal-Wallis	17	12	0	29 (4.2%)
Linear regression	11	14	2	27 (3.9%)
t-test	13	11	0	24 (3.4%)
Fisher exact test	16	8	0	24 (3.4%)
Other ^2^	39	22	1	62 (8.9%)
ND ^1^	10	6	0	16 (2.3%)
**Statistical test consistency between Methods and Results sections**				
Perfect Agreement	155	127	13	295 (42.2%)
Name of test not reminded in Results	169	191	25	385 (55.2%)
Obvious discrepancy	10	8	0	18 (2.6%)

AoO: Annals of Oncology; BjC: British Journal of Cancer; BMJ: British Medical Journal.

^1^ ND: Not Described in the Methods section.

^2^Other: kappa test, ANOVA test, Pearson correlation test, Spearman test.

For the interventional studies, the test chosen was mainly parametric (89 studies, 69.5% of cases). The tests most frequently used were: chi-square test (21 studies, 16.4%), logistic regression (19 studies, 14.8%), t-test (16 studies, 12.5%). A perfect consistency of reporting for statistical test between Methods and Results sections was observed for 43 studies (33.6% of cases). Name of test was not reminded in Results section for 80 studies (62.5% of cases) and obvious discrepancy for test reporting was noted in 5 studies (3.9%, Table 3).

**Table 3:** Reporting of variables and statistical test in interventional studies (N = 128).

Reporting Item	AoO (N = 67)	BjC (N = 52)	BMJ (N = 9)	Total, N (%)
**Primary endpoint described in Methods**				
Yes	56	44	9	109 (85.2)
No	11	8	0	19 (14.8)
**Significant results for primary endpoint**				
Yes	38	41	8	87 (68)
No	25	9	1	35 (27.3)
NA^1^	4	2	0	6 (4.7)
**Concordant conclusion on the main result**				
Yes	64	52	9	125 (97.7)
No	3	0	0	3 (2.3)
**Test described in Methods**				
Yes	62	49	9	120 (93.8)
No	5	3	0	8 (6.2)
**Type of test**				
Parametric	44	38	7	89 (69.5)
Nonparametric	18	11	2	31 (24.2)
ND^2^	5	3	0	8 (6.3)
**Name of test**				
Chi-square	12	7	2	21 (16.4)
Logistic regression	10	8	1	19 (14.8)
t-test	9	7	0	16 (12.5)
Wilcoxon / Kruskal-Wallis	6	6	1	13 (10.2)
Fisher exact test	7	5	1	13 (10.2)
Linear regression	2	8	2	12 (9.4)
Poisson regression	1	0	2	3 (2.3)
Other^3^	15	8	0	23 (18)
ND^2^	5	3	0	8 (6.2)
**Statistical test consistency between Methods and Results sections**				
Perfect Agreement	21	19	3	43 (33.6)
Name of test not reminded in Results	42	32	6	80 (62.5)
Obvious discrepancy	4	1	0	5 (3.9)

AoO: Annals of Oncology; BjC: British Journal of Cancer; BMJ: British Medical Journal.

^1^ NA: Not Assessable.

^2^ ND: Not Described in the Methods section.

^3^ Other: binomial test, ANCOVA test, ANOVA test, Pearson correlation test.

### Discrepancy in statistical tests (qualitative assessment)

Among the 23 articles classified in the “discrepancy in statistical methods reporting” groups, no articles were published in the BMJ. 8 articles were published in BJC and 15 were published in AoO. Table 4 reports the causes of discrepancy for observational studies. To illustrate the different types of discrepancies encountered in these articles, 2 types of discrepancies examples are given. First type: for some articles the tests were mentioned neither in the Methods section nor in the Results section. Second Type: tests reported in Methods section could not have given results presented in Results section (In one article method section reported solely that comparisons between groups were made using chi-square statistics. But in results Odds Ratio with a 95%CI were given).

**Table 4:** Description of causes of discrepancy for statistical tests in observational studies (N = 18).

Causes	Number of articles	Journals
tests only described in results	6	2 BJC, 4 AoO
Tests described in methods and results, but a test in results is added (unexcpected?)	3	3 AoO
Tests described neither in methods nor in results	4	2 BJC, 2 AoO
Tests described neither in methods nor in results, but cited in a reference (in S1 File or bibliography)	2	1 BJC, 1 AoO
Test reported in methods and results is eventually the same but reported with two different names (eg: Mann Whitney vs Wilcoxon)	1	1 AoO
Test reported in methods does not allow to get the results reported	1	1 AoO
Test described only in methods section	1	1 BJC

AoO: Annals of Oncology; BJC: British Journal of Cancer.

### Factors associated to discrepancy in statistical tests reporting

338 studies (40.9%) were included in group “1” and 488 studies (59.1%) were classed in group “0”. In univariable analysis, the three following trial characteristics were associated with perfect consistency for statistical test, with a p. value ≤ 0.2: Sample size, Type of study and Name of journal (Table 5).

**Table 5:** Analysis by univariable logistic regression of perfect statistical tests consistency for selected variables.

Variable	Univariable Analysis	
	OR (95%CI)	p value^1^
**Journal**		0.2
*Annals of Oncology*	1	
*British Journal of Cancer*	0.8 [0.6–1.07]	
*British Medical Journal*	0.66 [0.35–1.24]	
**Year of publication**		0.452
2009	1	
2010	1.18 [0.72–1.93]	
2011	0.82 [0.51–1.33]	
2012	1.03 [0.65–1.62]	
2013	0.86 [0.54–1.38]	
2014	0.64 [0.31–1.32]	
**Sample size, n° of patients**		0.005
Grp1*:10≤ n< 160	1	
Grp2*:160≤ n< 2.57*10^3^	0.92 [0.66–1.29]	
Grp3*:2.57*10^3^≤ n< 1.09*10^7^	0.55 [0.37–0.82]	
**Tumor Site**		0.42
Breast	1	
Colon/Rectum	1.02 [0.61–1.71]	
Gynecological	1.03 [0.56–1.9]	
Leukemia/Lymphoma	1.69 [0.91–3.14]	
Prostate	1.69 [0.85–5.36]	
Lung	1.3 [0.65–2.63]	
Stomach/Oesophagus	1.81 [0.82–3.98]	
Renal	0.96 [0.38–2.42]	
Pancreas	2.32 [0.89–6.05]	
Head and Neck	2.11 [0.79–5.6]	
Other	1.08 [0.65–1.79]	
**Type of study**		0.132
Observational	1	
Interventional	0.75 [0.48–1.16]	
Phase III RCT^2^	0.52 [0.23–1.19]	
**Concordant conclusion on the MEP**^3^ **result**		0.245
No	1	
Yes	1.93 [0.61–6.1]	
**Type of test**		0.43
Parametric	1	
Nonparametric	0.83 [0.54–1.28]	
**MEP**^3^ **described in Methods**		0.92
No	1	
Yes	0.95 [0.38–2.39]	

* Group 1,2 and 3.

^1^ P value calculated from Likelihood Ratio-test.

^2^ Phase III Randomised Controlled Trials.

^3^ MEP: Main End Point.

In multivariable analysis, Sample size (number of included patients), remained an independent factor associated with consistency: aOR (adjusted on Name of the Journal, Year of publication, tumor Site, Type of Study, Concordant Conclusion on the Main EndPoint, Type of Test and Main EndPoint described in methods) for group 3 compared to group 1 was equal to 0.58 [0.38–0.88] (P value = 0.014, LR-test, Table 6).

**Table 6:** Analysis by multivariate logistic regression of perfect statistical tests consistency.

N = 628	adj. OR(95%CI)	p value^1^
**Journal**		
Annals of Oncology	1	
British Medical Journal	0.8 (0.36,1.78)	0.468
British Journal of Cancer	0.8 (0.56,1.15)	
**Sample size, N° of patients**		
Grp1*: 10ze, N° o	1	**0.009**
Grp2*: 160≤6009: 10ze,	1.02 (0.67,1.54)	
Grp4*: 2.57*10^3^≤ n< 1.09*10^7^	0.52 (0.31,0.89)	
**Year of publication**		
2009	1	
2010	1.39 (0.77,2.48)	
2011	0.91 (0.52,1.6)	0.691
2012	1.1 (0.64,1.9)	
2013	0.99 (0.55,1.77)	
2014	0.84 (0.35,2.01)	
**Tumor Site**		
Breast	1	
Colon/Rectum	1.18 (0.68,2.03)	
Gynecological	1.27 (0.66,2.43)	0.253
Renal	1.01 (0.39,2.63)	
Leukemia/Lymphoma	1.63 (0.84,3.15)	
Lung	1.63 (0.77,3.46)	
Head and Neck	2.4 (0.87,6.58)	
Other	1.16 (0.68,1.98)	
Pancreas	3.08 (1.13,8.36)	
Prostate	2.16 (1.03,4.52)	
Stomach/Oesophagus	1.92 (0.85,4.34)	
**MEP described in Methods**		
No	1	
Yes	0.97 (0.28,3.39)	0.963
**Type of study**		
Observational	1	
Interventional	0.69 (0.38,1.23)	0.206
Phase III RCT	0.51 (0.19,1.35)	
**Type of test**		
Non-parametric	1	
Parametric	0.64 (0.38,1.06)	0.084
**Concordant conclusion on the MEP result**		
No	1	
Yes	1.4 (0.39,5.05)	0.61

* Group 1,2 and 3.

^1^ P value calculated from Likelihood Ratio-test.

## Discussion

Among all 826 articles in the present systematic review, 698 (84.5%) were observational studies and there was a good quality of reporting of variables and statistical tests in Methods parts (100% and 97.7% of cases, respectively). However, only 295 observational studies (42.2% of cases) had a perfect consistency of statistical tests between Methods and Results part. The same observations can be highlighted for interventional studies: A good reporting of the primary endpoint in 85.2% of cases and of the statistical test in the Methods section in 93.8% of cases. Similarly, a perfect consistency for statistical tests between methods and results sections was found in only 43 studies (33.6% of cases). This can be explained by a great proportion of studies, in which statistical tests are not clearly reported in Results section (54.9% of observational studies and 62.5% of interventional studies) and consistency cannot be assessed.

In fact, each journal establishes and publishes their specific requirements for data analysis, and there is no consensus for this aspect of peer review: Some journal editors currently request a statistical analysis of trial data by an independent biostatistician before accepting studies for publication. Others ask authors to say whether the study data are available to third parties to view and/or use/reanalyze, while still others encourage or require authors to share their data with others for review or reanalysis *(Recommendations for the Conduct*, *Reporting*, *Editing*, *and Publication of Scholarly work in Medical Journals*, *2013)*. Another element to be considered, is the poor communication, concerning statistical methods, between statistician(s) and leading author(s). Consequently, names of statistical tests utilised in Results section are inconsistently reported in publications. So, a significant proportion of studies lacked transparency for description of the statistical test in the Results section and this may be evidence of a gap in current reporting guidelines [4, 13]. STROBE and CONSORT statements recommend a detailed and complete description of the statistical test chosen for the primary endpoint in Methods section. However, nothing is said about reporting of a reminder of the statistical test used in Results section, and currently, readers of observational and interventional studies are not always able to verify the consistency for statistical test. Consequently, reproducibility of such studies cannot be assured. In literature, some articles and books have already demonstrated the importance of the reporting for statistical analysis and statistical results in high quality scientific publications [14,15]. We observed that in 18 observational studies (2.6%) tests announced were not performed. An explanation could be that although authors actually performed all analyses mentioned in the method section, they didn’t report all of them in the result section because results were of no interest. Yet such information and withdrawal decision should still be mentioned. The results of this study might suggest that a systematic statistical checking should be recommended during the reviewing process, in order to raise such discrepancy between tests reported in methods and results section.

The qualitative analysis of the discrepancies highlighted the possible failure of the peer review system: in some cases, simple CONSORT and STROBE recommendations have not been followed in reporting (tests reported in statistics, rather than in methods section), in other cases, some rough mistakes have not been corrected by an attentive reviewing. In conclusion, statistical aspects of studies in oncology could be more carefully described before submission for publication and adding of a statistical reviewer in medical journals, which improve quality of reporting [16], should be mandatory.

Results of the multivariable analysis have revealed that the sample size (number of patients included) is an independent factor associated with perfect statistical test consistency (p = 0.009). In studies with small sample sizes, the parametric assumptions are not always applicable and, therefore, the authors pay particular attention to the types of statistical tests used (parametric or nonparametric) and report them more frequently, both in Methods section and in Results section. As regards to the statistical analyses section, 88.8% of observational studies and 69.5% of interventional studies used parametric tests, which gives us to understand that the majority of authors put forward a normal assumption for the distribution of their variables.

Our systematic review has several limitations. First, analysis focused on 3 journals. The results should therefore be confirmed, although selection of these three journals is fairly representative of literature in oncology. We must also remember that these results mainly concerns reporting of observational studies and we cannot draw any definite conclusion on included interventional studies (128 studies, 15.5%). A further work should be to explore whether the methodology used in those articles is appropriate, and in accordance with the actual statistical guidelines. Such work can only be undertaken with the help of a large sample of leading experts in statistics, in order to get to a consensual definition of appropriateness. Another issue raised by this review is the problem of the multiplicity in statistical tests performed in observational studies: we reported the frequency of studies where a significant association was found between exposure and disease but we did not measure in those studies the quantity of statistical multiplicity and thereby the risk of spurious conclusions it would have led to. The issue of statistical multiplicities in observational oncological studies remains to be address elsewhere.

In conclusion, in Methods section, our results show a 100% frequency of reporting for variables in observational studies and a 85% frequency for Primary Endpoint in interventional studies. A discrepancy between tests reported in methods and results sections was identified in 23 articles, whose 18 were observational studies. Current guidelines, like STROBE or CONSORT, do not yet take into account this aspect of reporting and thus we encourage authors and peer reviewers to carefully verify consistency of statistical tests.

## Supporting Information

S1 File Articles extraction form data base. (XLS)

S2 File PRISMA checklist. (DOCX)
